# Reproducible Gut Microbiome Alterations in Major Depressive Disorder: A Systematic Review of Taxonomic and Functional Findings

**DOI:** 10.3390/epidemiologia7040104

**Published:** 2026-07-23

**Authors:** Gulshat Dalibayeva, Maya Goremykina, Samat Kozhakhmetov, Almagul Kushugulova, Alibek Kossumov, Sundetgali Kalmakhanov, Ainur Doszhan

**Affiliations:** 1 Faculty of Medicine and Health Care, Al-Farabi Kazakh National University, Almaty 050040, Kazakhstan; gdalibayeva@gmail.com (G.D.); sundetgali.kalmakhanov@gmail.com (S.K.); 2 Department of Internal Medicine and Rheumatology, Semey Medical University NJSC, Semey 071400, Kazakhstan; 3 National Laboratory Astana, Nazarbayev University, Astana 010000, Kazakhstan; sskozhakhmetov@gmail.com (S.K.); akushugulova@nu.edu.kz (A.K.); alibek.kossumov@nu.edu.kz (A.K.); 4 JSC National Scientific Medical Center, Astana 010009, Kazakhstan; ainurdoszhan8@gmail.com

**Keywords:** major depressive disorder, gut microbiome, microbiota–gut–brain axis, dysbiosis, metagenomics, depression, microbiome

## Abstract

Background/Objectives: Major depressive disorder (MDD) has been increasingly associated with alterations of the gut microbiome through the microbiota–gut–brain axis. However, published findings remain highly heterogeneous, limiting identification of reproducible microbial signatures associated with depression. This systematic review aimed to evaluate reproducible taxonomic and functional gut microbiome alterations in patients with MDD compared with healthy controls. Methods: A systematic literature search was conducted in PubMed/MEDLINE, Scopus, Web of Science Core Collection, and the Cochrane Library for studies published between January 2016 and December 2025. Observational human studies evaluating gut microbiome composition in adults with clinically diagnosed MDD and healthy control groups were included. Methodological quality was assessed using the Newcastle-Ottawa Scale. Due to substantial methodological heterogeneity, findings were synthesized using structured qualitative narrative analysis. Results: Sixteen observational studies were included in the qualitative synthesis. Findings related to alpha diversity were inconsistent across studies, whereas beta diversity alterations demonstrated greater reproducibility across independent cohorts. The most recurrent microbiome pattern involved depletion of short-chain fatty acid (SCFA)-producing bacteria, particularly Faecalibacterium and Roseburia, together with recurrent alterations affecting members of the Ruminococcaceae, Lachnospiraceae, and Clostridia groups. Functional microbiome alterations demonstrated greater consistency than higher-level taxonomic findings and included reduced butyrate synthesis pathways, dysregulated amino acid and tryptophan metabolism, increased lipopolysaccharide biosynthesis, and enrichment of pro-inflammatory microbial signatures. Antidepressant-naïve cohorts generally demonstrated more homogeneous dysbiosis patterns than mixed-treated populations. Conclusions: Current evidence suggests that functional gut microbiome dysregulation may represent a more reproducible biological feature of MDD than isolated taxonomic alterations alone. However, substantial heterogeneity in study design, participant characteristics, sequencing methodologies, and analytical approaches continues to limit clinical translation. Large-scale longitudinal multi-omics studies using standardized methodologies are required to clarify the role of the gut microbiome in depressive disorders and to evaluate the potential utility of microbiome-based biomarkers and interventions in mental health and public health practice.

## 1. Introduction

Major depressive disorder (MDD) remains one of the leading contributors to the global burden of disease and disability worldwide [1]. According to the Global Burden of Disease Study 2021, depressive disorders affect approximately 332 million people globally, corresponding to nearly 4% of the world population [1]. Among adults, prevalence is estimated at approximately 5%, including 4% among men and 6% among women [1]. In addition to emotional and cognitive symptoms, depression is associated with impaired social functioning, reduced quality of life, decreased work productivity, increased healthcare utilization, and elevated mortality risk, including suicide and cardiovascular complications [1,2]. The burden of depressive disorders further increased during the COVID-19 pandemic, with an estimated 53.2 million additional cases globally in 2020 [2]. Despite the availability of pharmacological and psychotherapeutic interventions, treatment outcomes remain heterogeneous, and a substantial proportion of patients fail to achieve sustained remission [3].

Growing evidence suggests that depression is a multifactorial disorder involving interactions between neuroendocrine, metabolic, inflammatory, and environmental mechanisms [3,4,5]. In recent years, increasing attention has been directed toward the gut microbiome as a potentially important component in the pathophysiology of psychiatric disorders [4,5,6,7]. From a public health perspective, the gut microbiome represents a biologically dynamic and potentially modifiable system influenced by nutrition, lifestyle, medication exposure, environmental factors, and host physiology [5,6,7,8].

The gut microbiome consists of trillions of microorganisms involved in metabolic, endocrine, immune, and neural regulation [6,7]. Through the microbiota–gut–brain axis, intestinal microorganisms may influence central nervous system functioning through immune, endocrine, metabolic, and neural signaling pathways [5,6,7,8,9]. Experimental and clinical evidence suggests that gut microbial metabolites participate in the regulation of intestinal barrier integrity, inflammatory responses, neurotransmitter synthesis, and hypothalamic–pituitary-adrenal axis activity [8,9,10].

Short-chain fatty acids (SCFAs), particularly butyrate, acetate, and propionate, are among the most extensively studied microbial metabolites in gut–brain axis research [8,10]. SCFAs are produced through bacterial fermentation of dietary fiber and contribute to maintenance of epithelial barrier function and modulation of immune and inflammatory signaling [8,10]. Bacterial taxa belonging to the families *Ruminococcaceae* and *Lachnospiraceae* play a central role in SCFA production and intestinal homeostasis [10,11].

Accumulating evidence indicates that gut microbiota alterations are associated with multiple chronic diseases, including inflammatory bowel disease, irritable bowel syndrome, obesity, asthma, autoimmune disorders, neurodegenerative diseases, and psychiatric conditions [11,12,13,14,15,16,17]. Population-based microbiome studies have also demonstrated associations between gut microbial composition and mental health outcomes. In a microbiome-wide association study involving more than 7000 participants, several microbial taxa were significantly associated with depressive symptoms [18]. Similarly, depletion of the genera *Coprococcus* and *Dialister* was associated with lower quality of life and increased depressive symptom severity in a large population cohort study [19].

Over the past decade, numerous observational studies have investigated gut microbiota composition in patients with MDD. Several reports demonstrated reduced abundance of short-chain fatty acid–producing bacteria and increased representation of potentially pro-inflammatory taxa in depressive disorders [20,21,22,23,24]. Associations between gut microbiota composition and anxiety symptoms, sleep quality, and treatment response have also been described [20,21,22,23,24,25]. However, findings remain highly heterogeneous. Previous studies included substantially different populations, including patients with depression and anxiety disorders, individuals with subclinical depressive symptoms, pregnant women with psychological distress, and patients with bipolar disorder [20,21,22,23,24,25,26]. In addition, studies differed in diagnostic criteria, medication exposure, geographic regions, sequencing methodologies, and bioinformatic approaches [21,22,23,24,25,26,27].

As a result, reported alterations in gut microbiota composition remain inconsistent across studies, particularly at higher taxonomic levels. Findings involving the phyla *Firmicutes* and *Bacteroidetes*, as well as microbial diversity indices, have shown limited reproducibility [24,25,26,27,28]. Although previous systematic reviews and meta-analyses summarized general microbiome alterations in depression, many focused primarily on overall diversity measures or selected bacterial taxa without systematically evaluating reproducibility of taxonomic findings across studies [24,27,28,29].

From a public health perspective, identification of reproducible microbiome patterns associated with depression may contribute to improved understanding of disease mechanisms and support future development of microbiome-informed prevention, risk stratification, and therapeutic approaches. Given the rapid expansion of microbiome research and the substantial heterogeneity of published findings, an updated structured synthesis focused on reproducibility of taxonomic alterations is needed.

Therefore, the aim of this systematic review was to evaluate taxonomic alterations in the gut microbiome in patients with major depressive disorder compared with healthy controls, with particular emphasis on reproducibility of findings and sources of heterogeneity across studies.

## 2. Materials and Methods

This systematic review was conducted in accordance with the Preferred Reporting Items for Systematic Reviews and Meta-Analyses (PRISMA 2020) guidelines [30]. The review protocol was registered in the International Prospective Register of Systematic Reviews (PROSPERO) under registration number CRD420261410339 [31]. The completed PRISMA checklist is provided in the Supplementary Materials (Table S1).

The objective of the review was to evaluate reproducible taxonomic alterations in the gut microbiome among patients with major depressive disorder (MDD) compared with healthy controls and to assess potential sources of heterogeneity across studies.

### 2.1. Eligibility Criteria

Eligibility criteria were defined before the screening process to ensure methodological consistency and minimize selection bias during study inclusion (Table 1).

The eligibility criteria were developed to ensure inclusion of clinically comparable studies evaluating gut microbiome composition specifically in MDD. Restricting the population to adults with depressive disorders reduced heterogeneity associated with pediatric microbiome development and severe psychiatric comorbidities. Studies focused primarily on bipolar disorder, schizophrenia, or neurological diseases were excluded because microbiome alterations may substantially differ across psychiatric and neurodegenerative conditions.

Only studies including healthy control groups were considered eligible in order to allow direct comparison of taxonomic composition between patients with depression and non-depressed individuals. Experimental animal studies were excluded because their findings are not directly comparable to human observational microbiome data.

The publication period was limited to studies published between 2016 and 2025 to reflect contemporary microbiome sequencing technologies and current bioinformatic approaches. Earlier microbiome studies frequently used limited sequencing depth and outdated taxonomic classification pipelines, reducing comparability with more recent investigations.

### 2.2. Search Strategy

A comprehensive electronic literature search was conducted in PubMed/MEDLINE, Scopus, Web of Science Core Collection, and the Cochrane Library to identify studies evaluating gut microbiota alterations in patients with major depressive disorder. The search included publications from 1 January 2016 to 31 December 2025. The final electronic database search was conducted on 15 January 2026.

The search strategy was developed using combinations of controlled vocabulary terms and free-text keywords related to depressive disorders and gut microbiota. Boolean operators (“AND”, “OR”) were applied to maximize sensitivity while maintaining specificity for observational human studies. Search strategies were adapted according to the indexing systems and technical requirements of each database (Table 2).

Overall, 1795 records were identified across all databases. After removal of 598 duplicate records, 1197 publications remained for title and abstract screening.

During the initial screening stage, 1098 records were excluded because they were unrelated to major depressive disorder, did not evaluate gut microbiota composition, represented animal or experimental studies, or were review articles, conference abstracts, editorials, or non-original publications.

**Figure 1:**
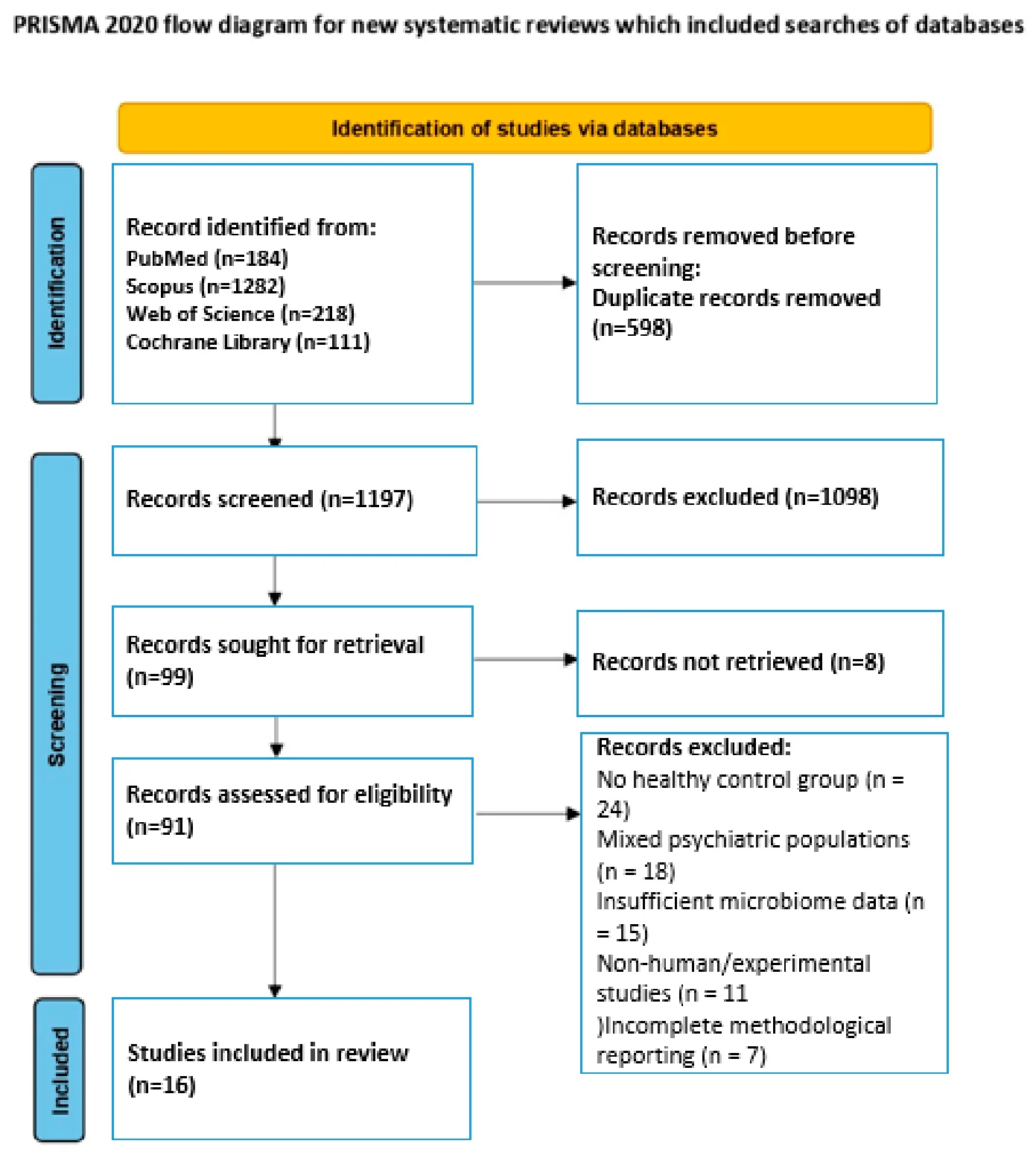
The detailed study selection process is presented in the PRISMA 2020 [30] flow diagram.

A total of 99 full-text articles were assessed for eligibility. Of these, 83 studies were excluded after full-text review for the following reasons: absence of healthy control group (*n* = 24); mixed psychiatric populations without separate MDD analysis (*n* = 18); insufficient taxonomic microbiome data (*n* = 15); non-human or experimental design (*n* = 11); duplicate cohorts or overlapping datasets not retrieved (*n* = 8); incomplete methodological reporting (*n* = 7). Ultimately, 16 studies met all eligibility criteria and were included in the qualitative synthesis. The detailed study selection process is presented in the PRISMA 2020 [30] flow diagram (Figure 1).

### 2.3. Study Selection and Data Extraction

Study selection was independently performed by two reviewers using a two-stage screening process. During the first stage, titles and abstracts were evaluated to identify potentially relevant publications. Studies meeting the eligibility criteria or those with uncertain eligibility based on abstract review underwent full-text assessment.

Full-text articles were independently reviewed by both investigators. Disagreements regarding study eligibility were resolved through discussion and consensus. When consensus could not be reached, a third reviewer was consulted.

Data extraction was performed using a standardized extraction form developed prior to analysis. Information collected from each study included publication year, country, study design, sample size, demographic characteristics, diagnostic criteria for MDD, medication exposure, microbiome sequencing methodology, diversity measures, reported taxonomic alterations, and functional or metabolic findings. Particular attention was given to reproducibility of bacterial taxa across studies and to factors potentially contributing to heterogeneity, including geographic region, sequencing platform, and population characteristics.

#### Definition of Reproducibility

To improve interpretability and consistency of qualitative synthesis, reproducibility of microbiome findings was evaluated based on the frequency and directionality of reported taxonomic alterations across included studies. Findings reported in ≥70% of studies with consistent directionality were interpreted as highly reproducible, whereas findings identified in 40–69% of studies were considered moderately reproducible. Alterations reported inconsistently or in fewer than 40% of studies were classified as having low reproducibility.

Both taxonomic and functional microbiome findings were evaluated using this structured narrative framework. Particular attention was given to short-chain fatty acid–producing bacteria, inflammatory-associated taxa, and microbial metabolic pathways repeatedly identified across independent cohorts. Because of substantial heterogeneity in sequencing methodologies, bioinformatic pipelines, participant characteristics, and statistical reporting, reproducibility assessment was intended to identify general consistency of findings rather than establish quantitative pooled effect estimates.

### 2.4. Risk-of-Bias Assessment

Methodological quality of the included studies was evaluated using the Newcastle–Ottawa Scale (NOS), a validated tool commonly used for assessment of observational studies included in systematic reviews (Table 3).

The NOS evaluates studies across three domains: selection of study participants, comparability of study groups, and adequacy of exposure or outcome assessment. Studies scoring 7–9 points were considered low risk of bias, studies scoring 5–6 points were classified as moderate risk, and studies scoring ≤ 4 points were considered high risk of bias. Quality assessment was independently conducted by two reviewers.

Overall, methodological quality was moderate to high across the included studies. Lower NOS scores were primarily attributable to small sample sizes, limited adjustment for potential confounding factors, mixed antidepressant exposure, and restricted population representativeness. Although microbiome sequencing methodologies were generally well described, the predominance of observational designs limits causal inference regarding the relationship between gut microbiota alterations and major depressive disorder.

### 2.5. Data Synthesis

A formal meta-analysis was not performed because substantial methodological and clinical heterogeneity was identified across the included studies. Important differences were observed in participant characteristics, diagnostic criteria, antidepressant exposure, sequencing methodologies, taxonomic classification pipelines, and reporting of microbiome outcomes.

Pooling these heterogeneous data into a single quantitative estimate could therefore produce potentially misleading conclusions. The heterogeneity extended beyond effect size variability and involved substantial differences in study populations, diagnostic criteria, antidepressant exposure, sequencing technologies, taxonomic resolution, bioinformatic pipelines, and reported microbiome outcomes, precluding calculation of meaningful pooled effect estimates or formal heterogeneity statistics such as I^2^. Similarly, subgroup analyses were considered inappropriate because of the limited number of studies within individual methodological strata.

Taxa were grouped according to consistency of reported findings, with particular attention to repeatedly identified short-chain fatty acid-producing bacteria and inflammation-associated microbial taxa. Descriptive frequencies of recurrent bacterial alterations were additionally calculated to identify the most reproducible microbiome patterns associated with major depressive disorder.

To facilitate interpretation of recurrent findings, a semi-quantitative assessment of reproducibility was performed. For each microbial taxon, studies were categorized according to the reported direction of change (decreased abundance, increased abundance, no significant difference, or inconsistent findings). Reproducibility categories were defined a priori for the present review and were used solely as descriptive indicators of consistency across independent studies. Only taxa reported in at least three independent studies were included in the reproducibility matrix.

## 3. Results

### 3.1. Characteristics of Included Studies

The final qualitative synthesis included 16 observational studies published between 2019 and 2025. Fourteen studies used a case–control design [32,33,34,36,37,38,40,41,42,43,44,45,46,47], whereas two studies applied cross-sectional analytical approaches [35,39] (Table 4).

Most investigations originated from China (*n* = 10) [33,34,35,37,38,39,40,42,43,44,45], followed by the United States (*n* = 2) [32,41], Taiwan (*n* = 1) [46], Italy (*n* = 1) [47], Russia (*n* = 1) [36], and one multicenter international cohort [39].

Depression was assessed using DSM-IV, DSM-5, ICD-10 criteria, or validated depression rating scales. Antidepressant-naïve or drug-free participants were specifically evaluated in six studies [33,35,36,38,40,44], whereas the remaining investigations included mixed-treated or partially medicated cohorts [32,34,37,41,45,46,47].

Microbiome analysis was predominantly based on 16S rRNA sequencing [32,33,38,41,42,43,44,45,46,47]. Shotgun metagenomic sequencing was applied in four studies [34,35,36,37], and recent investigations increasingly incorporated multi-omics and metabolomic integration approaches [39,40,44].

Several studies additionally evaluated specific clinical correlates, including inflammatory biomarkers [32,44], cognitive dysfunction [33], sleep quality [43], anxiety symptoms [41], postpartum depression [45], and neuroimaging associations [46].

### 3.2. Diversity Findings

Findings related to alpha diversity demonstrated substantial heterogeneity across the included studies. Several investigations reported reduced microbial richness and community evenness in patients with MDD [32,33,34,35,38,40,43,44], whereas others observed no significant differences between depressive and control cohorts [36,37,41,45,46]. Increased alpha diversity was reported only occasionally and was generally limited to smaller cohorts [42,47].

In contrast, alterations in beta diversity were more consistently identified across studies. Multiple investigations demonstrated significant differences in overall microbial community composition between patients with MDD and healthy controls [32,33,34,35,36,37,38,39,40,42,43,44]. These findings were observed across geographically distinct populations and different sequencing platforms, suggesting that compositional restructuring of the gut microbiome may represent a more reproducible feature of depression than alpha diversity measures alone.

Importantly, significant beta diversity separation was also observed in several antidepressant-naïve cohorts [33,35,36,38,40,44], indicating that microbiome alterations cannot be explained solely by psychopharmacological treatment exposure.

Overall, the available evidence suggests that community composition (beta diversity) demonstrates greater consistency across studies than within-sample diversity measures (alpha diversity), which remain highly heterogeneous among MDD cohorts (Table 5).

### 3.3. Reproducible Taxonomic Alterations

Across the included studies, the most consistently reported microbial alterations involved depletion of short-chain fatty acid-producing taxa. Reduced abundance of *Faecalibacterium* was identified in seven studies, representing the most reproducible taxonomic finding associated with major depressive disorder [32,33,34,36,41,42,45]. Several investigations additionally linked lower *Faecalibacterium* abundance to increased depressive symptom severity, inflammatory dysregulation, and impaired microbial metabolic activity (Table 6).

Reduced abundance of *Roseburia*, another major butyrate-producing genus, was reported in five studies [33,34,36,42,45]. Similarly, decreased abundance of members of the family *Ruminococcaceae* was observed in five studies [32,34,41,42,44], while depletion of taxa belonging to the class *Clostridia* was identified in five studies [32,33,34,36,41]. Reduced abundance of *Lachnospiraceae* was reported in three studies [34,44,45].

Collectively, these findings indicate a recurrent loss of beneficial anaerobic microorganisms involved in fermentation of dietary fiber and production of SCFAs, particularly butyrate. Given the established role of butyrate in maintaining intestinal barrier integrity, modulating immune responses, and facilitating gut–brain communication, depletion of SCFA-producing bacteria may represent a biologically meaningful microbial signature of MDD.

**Figure 2:**
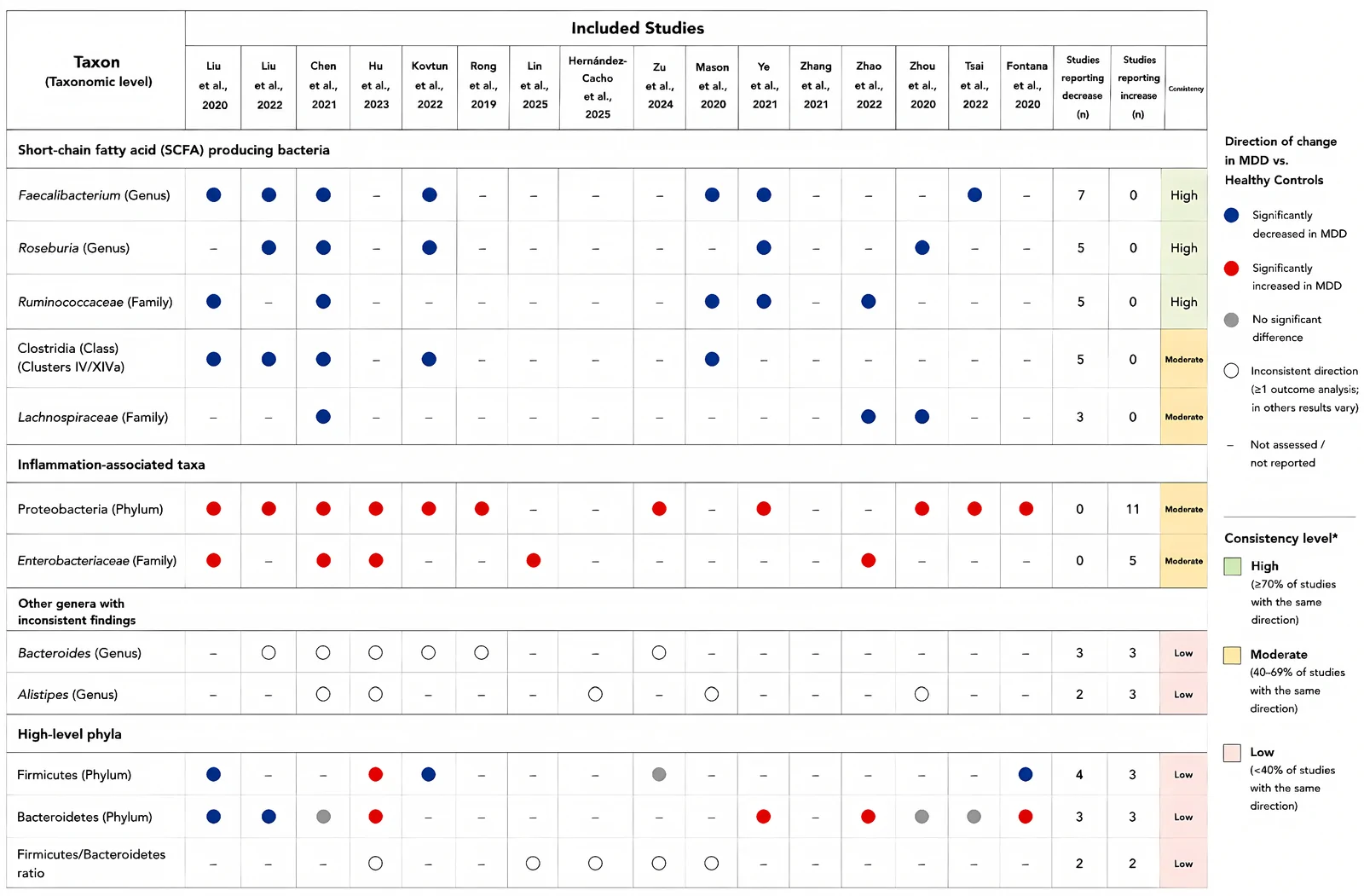
Reproducibility of taxonomic alterations reported across studies of major depressive disorder [32,33,34,35,36,37,38,39,40,41,42,43,44,45,46,47]. * Consistency level was assigned using a semiquantitative narrative synthesis approach based on the proportion of studies reporting concordant directions of change: high (≥75%), moderate (50–74%), and low (<50%).

In contrast, alterations involving higher taxonomic levels demonstrated substantial heterogeneity across studies. Increased abundance of Proteobacteria was reported in multiple independent cohorts [32,33,34,35,36,37,40,42,45,46,47], whereas findings regarding *Bacteroides*, *Alistipes*, *Firmicutes*, and the Firmicutes/Bacteroidetes ratio varied considerably between populations and study designs [35,39,40,45]. These observations suggest that functional alterations of microbial communities may be more reproducible than changes in individual taxa. A detailed study-by-study matrix of taxonomic alterations underlying the reproducibility analysis is presented in Supplementary Table S2 to facilitate interpretation of Figure 2 and ensure transparency of data synthesis.

The reproducibility and directionality of major taxonomic alterations identified across included studies are summarized in Figure 2. Reproducibility categories should be interpreted as indicators of consistency across independent studies rather than pooled quantitative effect estimates.

Each column represents an included study and each row represents a microbial taxon or taxonomic group. Blue circles indicate significantly decreased abundance in MDD, red circles indicate significantly increased abundance, gray circles indicate no significant difference, open circles indicate inconsistent findings, and dashes indicate that the taxon was not assessed or not reported. Summary columns show the number of studies reporting decreased or increased abundance and the corresponding reproducibility category. Reproducibility categories were assigned using a semi-quantitative narrative synthesis approach based on the proportion of studies reporting concordant directions of change.

### 3.4. Moderately Reproducible and Inconsistent Findings

Moderately reproducible alterations included increased abundance of inflammation-associated taxa, particularly Proteobacteria and members of the family Enterobacteriaceae, which were reported in several independent cohorts [34,35,42,44,47]. These taxa are commonly associated with microbial dysbiosis, intestinal barrier dysfunction, and systemic inflammatory activation.

In contrast, alterations involving Bacteroides [33,34,35,36,37,40], Alistipes [34,35,39,41,44], and Actinobacteria [34,39,40,44] demonstrated inconsistent directionality across studies. Some cohorts reported enrichment of these taxa in MDD, whereas others observed reduced abundance or no significant differences. Similar inconsistencies were observed across studies employing different sequencing platforms and analytical approaches.

At higher phylogenetic levels, reproducibility remained limited. Reported alterations involving Firmicutes and Bacteroidetes varied substantially between cohorts [32,33,34,35,36,37,38,39,40,41,42,43,44,45,46,47]. Likewise, the Firmicutes/Bacteroidetes ratio demonstrated inconsistent associations with depression and appeared highly dependent on study population characteristics, dietary factors, medication exposure, and analytical methodology (Table 7).

These findings indicate that broad taxonomic measures may be less informative than specific microbial taxa or functional microbial pathways when characterizing the gut microbiome in MDD.

### 3.5. Functional and Metabolic Alterations

Compared with taxonomic findings, functional microbiome alterations demonstrated greater reproducibility across studies.

Reduced pathways related to short-chain fatty acid biosynthesis, particularly butyrate metabolism, were reported in several metagenomic and multi-omics investigations [33,35,36,38,39,40,44]. Disturbances in amino acid metabolism and tryptophan-related pathways were additionally identified in multiple cohorts [34,38,39,40] (Table 8).

Recent large-scale studies demonstrated increased lipopolysaccharide biosynthesis pathways and enrichment of pro-inflammatory microbial metabolic signatures in patients with depression [35,38]. Multi-omics investigations additionally identified alterations in glycerophospholipid metabolism and multiple circulating metabolites associated with depressive symptom severity [39,40,44]. Functional findings included both directly measured metagenomic data and pathway inferences derived from multi-omics analytical approaches reported in the primary studies. Consequently, interpretation of functional alterations should take into account differences in sequencing technologies and analytical methodologies across studies.

Notably, several antidepressant-naïve cohorts demonstrated persistence of functional microbiome alterations independent of psychopharmacological treatment exposure [33,35,36,38,40,44]. These findings suggest that functional microbial dysregulation may represent an intrinsic feature of MDD rather than a secondary consequence of antidepressant treatment.

Overall, functional alterations related to SCFA metabolism, inflammatory signaling, and host-microbiome metabolic interactions appeared more consistent across studies than individual taxonomic findings.

### 3.6. Sources of Heterogeneity

Substantial heterogeneity was identified across included studies. Major sources of variability included geographic region, dietary patterns, antidepressant exposure, sequencing methodology, diagnostic criteria, microbiome bioinformatic pipelines, and differences in participant clinical characteristics.

Studies involving antidepressant-naïve participants generally demonstrated more homogeneous microbiome signatures than mixed-treated cohorts [33,35,36,38,40,44], suggesting a potential influence of psychopharmacological exposure on microbial composition. In contrast, mixed-treated cohorts frequently exhibited greater variability in reported taxonomic alterations [32,34,37,41,45,46,47].

Methodological heterogeneity additionally reflected differences in sequencing resolution. Earlier investigations primarily relied on 16S rRNA sequencing [32,33,41,42,43,44,45,46,47], whereas more recent studies increasingly incorporated shotgun metagenomics and multi-omics approaches [34,35,36,37,38,39,40,44], enabling functional characterization beyond taxonomic composition alone.

Overall, the reviewed evidence suggests that functional metabolic alterations demonstrate greater reproducibility than higher-level taxonomic composition across independent MDD cohorts.

## 4. Discussion

This systematic review evaluated reproducibility of gut microbiome alterations in major depressive disorder across observational human studies published between 2016 and 2025. Despite substantial taxonomic heterogeneity, several recurrent microbiome patterns were consistently identified across independent cohorts. The most reproducible alterations involved depletion of short-chain fatty acid-producing bacteria, particularly *Faecalibacterium*, *Roseburia*, and several members of the *Ruminococcaceae*, *Lachnospiraceae*, and *Clostridia* groups, although the frequency of individual taxonomic findings varied across studies.

A key observation of this review is that functional alterations appeared to demonstrate greater consistency across studies than taxonomic alterations. While individual bacterial taxa varied between cohorts, several studies consistently demonstrated disturbances in pathways related to butyrate synthesis, inflammatory signaling, amino acid metabolism, and lipopolysaccharide biosynthesis [33,35,36,38,39,40,44]. These observations suggest that microbial functional activity may represent a more robust biological correlate of depression than taxonomic composition alone.

The recurrent depletion of SCFA-producing bacteria may have important biological implications. SCFAs, particularly butyrate, contribute to maintenance of intestinal epithelial integrity, regulation of immune responses, modulation of neuroinflammatory signaling, and maintenance of gut barrier function [8,10]. Reduced abundance of butyrate-producing taxa may therefore contribute to increased intestinal permeability, systemic inflammation, and altered neuroimmune communication through the microbiota-gut–brain axis. Associations between reduced *Faecalibacterium* abundance and greater depressive symptom severity were additionally reported in several cohorts [33,35,38], supporting the potential clinical relevance of these alterations.

The present findings are generally consistent with previous systematic reviews reporting microbiome dysbiosis in depressive disorders [24,25,27,28,29]. Recent evidence syntheses also reported substantial heterogeneity of microbiome findings in depressive disorders while emphasizing the potential importance of inflammation-related and short-chain fatty acid-producing taxa [48,49,50,51,52]. A large meta-analysis by Gao et al. demonstrated inconsistent findings regarding alpha diversity and higher-level taxonomic composition across depression studies, highlighting substantial methodological variability between cohorts [48]. Contemporary reviews have further emphasized heterogeneity in microbiome findings while supporting the involvement of microbiota-gut-brain axis dysfunction, inflammatory pathways, and microbial metabolic disturbances in depression pathophysiology [49,50].

Recent reviews increasingly support the role of microbiota–gut–brain axis dysfunction in depression pathophysiology [50,51,52,53]. Current evidence suggests that inflammatory microbial pathways, microbial metabolites, and host-microbiome interactions may contribute to depression through immune activation, impaired intestinal barrier function, and neuroinflammatory signaling [50,51,52,53].

Recent microbiome-wide association studies and multi-omics investigations have additionally demonstrated associations between depressive symptoms and microbial metabolic signatures related to amino acid metabolism, bile acid pathways, short-chain fatty acid production, and inflammatory signaling [51,54,55,56]. Collectively, these findings support the concept that microbiome-associated metabolic dysfunction may represent a more reproducible feature of depression than individual taxonomic alterations.

Unlike previous reviews, the present study focused specifically on reproducibility of reported microbiome alterations across observational human studies and incorporated recent metagenomic and multi-omics investigations published after 2022 [48,54,55,56]. This approach enabled identification of microbial taxa and functional pathways demonstrating the greatest consistency across heterogeneous study populations and methodologies.

Antidepressant exposure emerged as an additional source of heterogeneity. Studies involving antidepressant-naive participants generally demonstrated more consistent microbiome alterations than mixed-treated cohorts [33,35,36,38,40,44]. This observation suggests that psychopharmacological treatment may substantially influence gut microbial composition and may partially contribute to inconsistent findings across the literature. At the same time, persistence of microbiome alterations in drug-naïve cohorts indicates that at least part of the observed dysbiosis may be associated with depressive pathology itself rather than medication effects alone.

Heterogeneity remains a central challenge in depression-related microbiome research. Geographic variation, dietary patterns, ethnicity, sequencing platforms, bioinformatic pipelines, and differences in diagnostic criteria likely contributed to variability in reported findings. Most included studies originated from East Asian populations, particularly China, which may additionally limit generalizability to other geographic and ethnic populations. Furthermore, several studies included relatively small cohorts, increasing the probability of unstable taxonomic estimates and limited reproducibility.

Important methodological variability was also observed across studies. Earlier investigations primarily relied on 16S rRNA sequencing, whereas more recent studies increasingly incorporated shotgun metagenomics and multi-omics approaches [34,35,36,37,38,39,40,44]. Although 16S sequencing remains widely used, it provides limited species-level and functional resolution compared with metagenomic analysis. Continued adoption of multi-omics approaches may improve both reproducibility and biological interpretation of microbiome findings in depression.

From a public health perspective, these findings have potential implications for prevention, risk stratification, and personalized intervention strategies. Depression represents a major contributor to disability and healthcare burden worldwide [1,2], while the gut microbiome represents a potentially modifiable biological system influenced by diet, lifestyle, environmental exposures, and medication use [5,6,7,8]. Identification of reproducible microbiome patterns associated with depressive disorders may facilitate development of microbiome-informed prevention strategies and adjunctive therapeutic approaches. Nevertheless, current evidence remains insufficient to support clinical implementation of microbiome-based biomarkers or interventions, and substantial methodological standardization is still required before such approaches can be reliably integrated into psychiatric practice.

Future research should prioritize large multicenter cohorts, standardized sequencing and bioinformatic pipelines, comprehensive assessment of dietary and medication exposures, and longitudinal designs capable of evaluating temporal relationships between microbiome alterations and depressive symptoms. Integration of metagenomics, metabolomics, inflammatory biomarkers, and detailed clinical phenotyping may further improve understanding of biologically relevant microbiome signatures in depression.

### Strengths and Limitations

This review has several important strengths. First, it focused specifically on the reproducibility of gut microbiome findings in major depressive disorder rather than solely providing a descriptive summary of reported bacterial alterations. This approach enabled identification of microbial taxa and functional pathways that remained relatively consistent across independent cohorts despite substantial methodological heterogeneity. Second, the review included studies involving clinically diagnosed major depressive disorder or validated depressive symptom phenotypes together with healthy comparison groups, allowing a comprehensive evaluation of microbiome alterations across depressive populations. Third, the review incorporated recent metagenomic and multi-omics investigations published between 2016 and 2025, thereby reflecting contemporary advances in microbiome research and enabling assessment of both taxonomic and functional microbial changes.

Several limitations should also be considered. Most included studies employed observational case–control or cross-sectional designs, limiting causal inference regarding the relationship between gut microbiota alterations and depression. Accordingly, it remains uncertain whether the observed microbiome alterations represent causal mechanisms contributing to depression, downstream consequences of the disorder, or secondary effects related to treatment exposure and lifestyle factors. Substantial methodological heterogeneity was observed across studies, including differences in diagnostic criteria, participant characteristics, antidepressant exposure, sequencing technologies, bioinformatic pipelines, and reporting of microbiome outcomes. Sample sizes varied considerably, and several cohorts remained relatively small, potentially affecting the stability and reproducibility of reported taxonomic findings.

Differences in diet, body mass index, smoking habits, medication exposure, and comorbid conditions are recognized determinants of gut microbiome composition and may partially explain inter-study variability observed across included cohorts. Although many primary studies adjusted for some of these factors, the extent of adjustment varied substantially between studies and residual confounding cannot be excluded.

Another important limitation relates to the geographic distribution of the available evidence. Most included studies were conducted in East Asian populations, particularly in China, which may limit the generalizability of findings to populations with different dietary habits, environmental exposures, healthcare systems, and genetic backgrounds. In addition, a quantitative meta-analysis was not performed because of substantial clinical and methodological heterogeneity across studies. Instead, findings were synthesized using a structured narrative approach and a semi-quantitative assessment of reproducibility. Finally, publication bias cannot be excluded, particularly given the growing scientific interest in microbiome-related psychiatric research and the tendency for positive findings to be reported more frequently than null results.

## 5. Conclusions

The findings of this systematic review indicate that gut microbiome alterations are associated with major depressive disorder, although substantial heterogeneity remains across studies. The most reproducible microbiome patterns involved depletion of short-chain fatty acid -producing bacteria, particularly Faecalibacterium and Roseburia, together with recurrent alterations affecting members of the Ruminococcaceae, Lachnospiraceae, and Clostridia groups. These findings suggest a common pattern of disruption among microbial communities involved in butyrate production and maintenance of gut microbial homeostasis.

Compared with taxonomic findings, functional microbiome alterations appeared to demonstrate greater consistency across studies. Disturbances in pathways related to butyrate metabolism, inflammatory signaling, lipopolysaccharide biosynthesis, and amino acid metabolism were repeatedly identified across independent cohorts, suggesting that microbial functional dysregulation may represent a more reproducible biological feature of depression than individual taxonomic alterations alone.

Despite these findings, methodological heterogeneity, limited standardization, geographic concentration of available evidence, and the predominance of observational study designs continue to restrict the clinical applicability of current evidence. Therefore, currently available data remain insufficient to support routine implementation of microbiome-based biomarkers or therapeutic interventions in psychiatric practice.

Future large-scale longitudinal studies integrating metagenomics, metabolomics, inflammatory biomarkers, and detailed clinical phenotyping are required to clarify causal relationships between gut microbiota and depression. Improved methodological standardization and multicenter validation will be essential for determining whether microbiome-informed prevention strategies, risk stratification tools, or adjunctive therapeutic approaches can contribute to future mental health and public health practice.

## Figures and Tables

**Table 1:** Eligibility criteria for study selection.

Category	Inclusion Criteria	Exclusion Criteria
Population	Adults with clinically diagnosed depressive disorders or validated depressive symptom phenotype	Bipolar disorder, schizophrenia, neurodegenerative diseases, pediatric populations
Comparison	Healthy control group	Absence of control group
Outcomes	Studies reporting gut microbiota composition or taxonomic alterations	Studies without microbiome sequencing data
Study design	Case–control, cohort, or cross-sectional observational studies	Case reports, editorials, conference abstracts, narrative reviews
Methodology	16S rRNA sequencing, shotgun metagenomics, whole metagenome sequencing, or multi-omics microbiome analysis	Animal or in vitro studies
Publication period	1 January 2016–31 December 2025	Studies published before 2016
Language	English	Non-English publications without accessible translation

**Table 2:** Search strategies and number of identified records.

Database	Search Strategy	Records Identified
PubMed/MEDLINE	(“major depressive disorder” [Title/Abstract] OR “depression” [Title/Abstract]) AND (“gut microbiota” [Title/Abstract] OR “gut microbiome” [Title/Abstract]) AND (case–control OR observational OR cohort) AND (humans [MeSH Terms])	184
Scopus	TITLE-ABS-KEY (“major depressive disorder” OR depression) AND TITLE-ABS-KEY (“gut microbiota” OR microbiome)	1282
Web of Science Core Collection	TS = (“major depressive disorder” OR depression) AND TS = (“gut microbiota” OR microbiome)	218
Cochrane Library	(“major depressive disorder” OR “depression”) AND (“gut microbiota” OR microbiome)	111

**Table 3:** Risk-of-bias assessment using the Newcastle–Ottawa Scale (NOS).

Study	Selection (0–4)	Comparability (0–2)	Exposure/Outcome (0–3)	Total Score	Risk of Bias	Main Limitation
Liu et al. [32]	3	1	3	7	Low	Mixed antidepressant exposure
Liu et al. [33]	3	1	2	6	Moderate	Limited dietary adjustment
Chen et al. [34]	4	2	3	9	Low	Female-only cohort
Hu et al. [35]	4	2	3	9	Low	Cross-sectional design
Kovtun et al. [36]	3	1	2	6	Moderate	Small sample size
Rong et al. [37]	3	1	2	6	Moderate	Heterogeneous mood-disorder population
Lin et al. [38]	4	2	3	9	Low	Cross-sectional design
Hernández-Cacho et al. [39]	4	2	3	9	Low	Depression phenotype-based classification
Zu et al. [40]	4	2	3	9	Low	Moderate sample size
Mason et al. [41]	3	1	2	6	Moderate	Small control group
Ye et al. [42]	3	1	3	7	Low	Limited statistical power
Zhang et al. [43]	3	1	3	7	Low	Potential residual confounding
Zhao et al. [44]	4	2	3	9	Low	Small sample size
Zhou et al. [45]	3	1	2	6	Moderate	Postpartum-specific population
Tsai et al. [46]	3	1	3	7	Low	Single-center recruitment
Fontana et al. [47]	3	1	2	6	Moderate	Mixed treatment exposure

**Table 4:** Description of included studies.

Study	Country	Design	Participants (MDD/HC)	Diagnostic Criteria	Medication Status	Sequencing Method	Main Clinical/Functional Focus
Liu et al., 2020 [32]	USA	Case–control	43/47	DSM	Mixed-treated cohort	16S rRNA	Inflammatory microbiome profile
Liu et al., 2022 [33]	China	Case–control	66/43	DSM-IV	Drug-naïve	16S rRNA	Cognitive dysfunction and inflammation
Chen et al., 2021 [34]	China	Case–control	62/46	DSM-5	Partially medicated	16S + shotgun metagenomics	Female-only MDD cohort
Hu et al., 2023 [35]	China	Cross-sectional	138/155	DSM-IV	Drug-naïve	Shotgun metagenomics	Large-scale taxonomic and functional analysis
Kovtun et al., 2022 [36]	Russia	Case–control	36/38	ICD-10	Drug-naïve	Whole metagenome sequencing	Functional pathway profiling
Rong et al., 2019 [37]	China	Case–control	31/30	DSM	Mixed-treated cohort	Shotgun metagenomics	Comparative mood-disorder microbiome analysis
Lin et al., 2025 [38]	China	Case–control	128/129	DSM-5	Antidepressant-naïve	16S rRNA	Depression Dysbiosis Index
Hernández-Cacho et al., 2025 [39]	Multicenter	Cross-sectional	400 total	Depression scale-based phenotype	Mixed exposure population	Multi-omics	Metabolomics and microbiome integration
Zu et al., 2024 [40]	China	Case–control	57/57	DSM	Drug-free	Multi-omics	Metabolic pathway alterations
Mason et al., 2020 [41]	USA	Case–control	52/10	DSM-IV	Mixed-treated cohort	16S rRNA	Anxiety symptom overlap
Ye et al., 2021 [42]	China	Case–control	26/28	DSM-IV	Pre-treatment	16S rRNA	Longitudinal microbiome dynamics
Zhang et al., 2021 [43]	China	Case–control	36/45	ICD-10	Mostly drug-naïve	16S rRNA	Sleep quality associations
Zhao et al., 2022 [44]	China	Case–control	24/26	DSM-5	Drug-naïve	Multi-omics	Cytokine-associated microbial alterations
Zhou et al., 2020 [45]	China	Case–control	28/16	DSM	Mixed-treated cohort	16S rRNA	Postpartum depressive symptoms
Tsai et al., 2022 [46]	Taiwan	Case–control	36/17	DSM	Mixed-treated cohort	16S rRNA + MRI	Brain–gut microbiome associations
Fontana et al., 2020 [47]	Italy	Case–control	34/20	DSM	Mixed-treated cohort	16S rRNA	Treatment-response microbiome analysis

**Table 5:** Diversity findings across included studies.

Diversity Parameter	Studies Reporting Finding	Representative Studies	Main Observation
Reduced alpha diversity	8	[32,33,34,35,38,39,40,44]	Lower microbial richness and evenness reported in a subset of MDD cohorts
No significant alpha diversity differences	6	[36,37,41,43,45,46]	Considerable heterogeneity across cohorts
Increased alpha diversity	2	[42,47]	Observed only in a limited number of cohorts
Significant beta diversity alterations	12	[32,33,34,35,36,37,38,39,40,42,43,44]	Reproducible compositional separation between MDD and healthy controls
Significant beta diversity in antidepressant-naïve cohorts	[33,35,36,38,40,44]	Evidence independent of treatment exposure	Persistent microbiome alterations in untreated MDD

**Table 6:** Most reproducible taxonomic alterations in depression.

Taxon	Direction of Change	Studies Reporting Concordant Alteration (*n*)	References
Faecalibacterium	Decreased	7	[32,33,34,36,41,42,45]
Roseburia	Decreased	5	[33,34,36,42,45]
Ruminococcaceae	Decreased	5	[32,34,41,42,44]
Clostridia	Decreased	5	[32,33,34,36,41]
Lachnospiraceae	Decreased	3	[34,44,45]
Proteobacteria	Increased	Frequently reported	[32,33,34,35,36,37,40,42,45,46,47]
Enterobacteriaceae	Increased	Frequently reported	[32,34,35,38,44]
Bacteroides	Variable	Inconsistent	[33,34,35,36,37,40]
Alistipes	Variable	Inconsistent	[34,35,39,41,44]
Firmicutes/Bacteroidetes ratio	Variable	Inconsistent	[35,39,40]

**Table 7:** Moderately reproducible and inconsistent microbiome findings.

Taxon/Parameter	Direction	Studies Reporting Finding, *n*	Representative Studies	Interpretation
Proteobacteria	↑	11	[32,33,34,35,36,37,40,42,45,46,47]	Marker of inflammatory dysbiosis
Enterobacteriaceae	↑	5	[32,34,35,38,44]	Pro-inflammatory association
Bacteroides	↑/↓	6	[33,34,35,36,37,40]	Population-dependent findings
Alistipes	↑/↓	5	[34,35,39,41,44]	Contradictory reproducibility
Actinobacteria	↑/↓	4	[34,39,40,44]	Inconsistent directionality
Firmicutes	Variable	Multiple studies	[32,33,34,35,36,37,38,39,40]	Poor reproducibility
Bacteroidetes	Variable	Multiple studies	[33,34,35,36,37,38,39,40]	Poor reproducibility
Firmicutes/Bacteroidetes ratio	Variable	Multiple studies	[32,33,34,35,36,37]	Limited reliability

Note: ↑, increased abundance; ↑/↓, inconsistent findings; Variable, no consistent direction of change across studies.

**Table 8:** Functional and metabolic microbiome alterations in MDD.

Functional Domain	Main Finding	Studies	Biological Relevance
SCFA metabolism	Reduced butyrate synthesis pathways	[33,35,36,38,39,40,44]	Reduced anti-inflammatory signaling and impaired gut–brain communication
Inflammatory pathways	Increased pro-inflammatory microbial signatures	[32,35,38,44]	Immune activation and chronic low-grade inflammation
Lipid metabolism	Altered glycerophospholipid metabolism	[39,40,44]	Membrane dysfunction and altered cellular signaling
Amino acid metabolism	Dysregulated tryptophan-related pathways	[34,38,39,40]	Altered neurotransmitter synthesis and neuroimmune regulation
LPS biosynthesis	Increased endotoxin-associated pathways	[35,38]	Intestinal inflammatory activation and systemic immune stimulation

## Data Availability

The original contributions presented in this study are included in the Article/Supplementary Materials. Further inquiries can be directed to the corresponding author.

## Supplementary Materials

The following supporting information can be downloaded at: https://www.mdpi.com/article/10.3390/epidemiologia7040104/s1, Table S1: The PRISMA checklist; Table S2: Study-by-Study Matrix of Taxonomic Alterations Included in the Reproducibility Analysis.
