# Supplementary material for: Reproducible Gut Microbiome Alterations in Major Depressive Disorder: A Systematic Review of Taxonomic and Functional Findings
Source: Epidemiologia (Basel). 2026 Jul 23;7(4):104. doi: 10.3390/epidemiologia7040104 (PMC13511779; doi:10.3390/epidemiologia7040104)
Supplement: Supplementary file 1 [file epidemiologia-07-00104-s001.zip › epidemiologia-4377714-supplementary.pdf]

Supplementary Table S1. PRISMA 2020 Checklist

Section and Topic	Item #	Checklist item	Location where item is reported
**TITLE**			
**Title**	1	Identify the report as a systematic review.	Title page
**ABSTRACT**			
**Abstract**	2	See the PRISMA 2020 for Abstracts checklist.	Abstract
**INTRODUCTION**			
**Rationale**	3	Describe the rationale for the review in the context of existing knowledge.	Introduction
**Objectives**	4	Provide an explicit statement of the objective(s) or question(s) the review addresses.	Introduction, final paragraph
**METHODS**			
**Eligibility criteria**	5	Specify the inclusion and exclusion criteria for the review and how studies were grouped for the syntheses.	Methods, Section 2.2; Table 1
**Information sources**	6	Specify all databases, registers, websites, organisations, reference lists and other sources searched or consulted to identify studies. Specify the date when each source was last searched or consulted.	Methods, Section 2.1
**Search strategy**	7	Present the full search strategies for all databases, registers and websites, including any filters and limits used.	Methods, Section 2.1; Table 2
**Selection process**	8	Specify the methods used to decide whether a study met the inclusion criteria of the review, including how many reviewers screened each record and each report retrieved, whether they worked independently, and details of automation tools if used.	Methods, Section 2.3
**Data collection process**	9	Specify the methods used to collect data from reports, including how many reviewers collected data from each report, whether they worked independently, and details of any verification procedures.	Methods, Section 2.3

**Data items**	10a	List and define all outcomes for which data were sought.	Methods, Section 2.3
	10b	List and define all other variables for which data were sought and describe assumptions made regarding missing information.	Methods, Section 2.3
**Study risk of bias assessment**	11	Specify the methods used to assess risk of bias in the included studies.	Methods, Section 2.4; Table 3
**Effect measures**	12	Specify for each outcome the effect measure(s) used in the synthesis or presentation of results.	Not applicable (narrative synthesis only)
**Synthesis methods**	13a	Describe the processes used to decide which studies were eligible for each synthesis.	Methods, Section 2.5
	13b	Describe any methods required to prepare the data for presentation or synthesis.	Not applicable
	13c	Describe any methods used to tabulate or visually display results of individual studies and syntheses.	Results, Tables 4–8 and Figure 2
	13d	Describe any methods used to synthesize results and provide a rationale for the choice(s).	Methods, Section 2.5 (structured narrative synthesis and reproducibility assessment)
	13e	Describe any methods used to explore possible causes of heterogeneity among study results.	Results, Section 3.6
	13f	Describe any sensitivity analyses conducted to assess robustness of the synthesized results.	Not conducted
**Reporting bias assessment**	14	Describe any methods used to assess risk of bias due to missing results in a synthesis.	Not formally assessed because quantitative synthesis was not performed
**Certainty assessment**	15	Describe any methods used to assess certainty (or confidence) in the body of evidence for an outcome.	Not assessed
**RESULTS**			
**Study selection**	16a	Describe the results of the search and selection process from identification to inclusion, ideally using a flow diagram.	Results, Section 3.1; Figure 1

	16b	Cite studies that might appear to meet the inclusion criteria but were excluded, and explain why they were excluded.	Figure 1
**Study characteristics**	17	Cite each included study and present its characteristics.	Results, Section 3.1; Table 4
**Risk of bias in studies**	18	Present assessments of risk of bias for each included study.	Results, Table 3
**Results of individual studies**	19	For all outcomes, present summary results for each included study using structured tables or figures.	Results, Tables 4–8 and Figure 2
**Results of syntheses**	20a	For each synthesis, summarise the characteristics and risk of bias among contributing studies.	Results, Sections 3.2–3.6
	20b	Present results of all statistical syntheses conducted.	Not applicable (no quantitative meta-analysis performed)
	20c	Present results of investigations of possible causes of heterogeneity among study results.	Results, Section 3.6
	20d	Present results of sensitivity analyses conducted to assess robustness of synthesized results.	Not conducted
**Reporting biases**	21	Present assessments of risk of bias due to missing results in a synthesis.	Not formally assessed because quantitative synthesis was not performed
**Certainty of evidence**	22	Present assessments of certainty (or confidence) in the body of evidence for each outcome assessed.	Not assessed
**DISCUSSION**			
**Discussion**	23a	Provide a general interpretation of the results in the context of other evidence.	Discussion, Section 4
	23b	Discuss limitations of the evidence included in the review.	Discussion, Strengths and Limitations
	23c	Discuss limitations of the review processes used.	Discussion, Strengths and Limitations
	23d	Discuss implications of the results for practice, policy, and future research.	Conclusions; Future Research Directions
**OTHER INFORMATION**			
**Registration and protocol**	24a	Provide registration information for the review, including register name and	Methods, Section 2.1 (Protocol submitted to

		registration number, or state that the review was not registered.	PROSPERO; registration pending)
	24b	Indicate where the review protocol can be accessed, or state that a protocol was not prepared.	Methods, Section 2.1
	24c	Describe and explain any amendments to information provided at registration or in the protocol.	No amendments reported
**Support**	25	Describe sources of financial or non-financial support for the review and the role of the funders.	Funding Statement
**Competing interests**	26	Declare any competing interests of review authors.	Conflicts of Interest
**Availability of data, code and other materials**	27	Report which materials are publicly available and where they can be found.	Data Availability Statement

Supplementary Table S2. Study-by-Study Matrix of Taxonomic Alterations Included in the Reproducibility Analysis

**Study**	**Faecalibacterium**	**Roseburia**	**Ruminococcaceae**	**Lachnospiraceae**	**Clostridia**	**Proteobacteria**
**Liu 2020 [1]**	↓	↓	↓	↓	↓	↑
**Liu 2022 [2]**	↓	↓	↓	↓	↓	↑
**Chen 2021 [3]**	↓	↓	↓	↓	↓	↑
**Hu 2023 [4]**	○	○	○	○	○	↑
**Kovtun 2022 [5]**	↓	↓	○	↓	↓	↑
**Rong 2019 [6]**	↓	–	–	–	–	↑
**Lin 2025 [7]**	–	–	–	–	–	–
**Hernández-Cacho 2025 [8]**	○	○	○	○	○	○
**Zu 2024 [9]**	○	○	○	○	○	↑

**Mason 2020 [10]**	↓	–	↓	–	↓	NS
**Ye 2021 [11]**	↓	↓	↓	–	↓	↑
**Zhang 2021 [12]**	–	NS	–	–	–	–
**Zhao 2022 [13]**	–	NS	–	↓	NS	NS
**Zhou 2020 [14]**	↓	–	–	↓	–	↑
**Tsai 2022 [15]**	–	NS	–	↓	NS	↑
**Fontana 2020 [16]**	–	NS	–	–	–	↑

↓ = decreased, ↑ = increased, NS = no significant difference, ○ = not reported, – = not assessed

References:

1. **.**Liu, R.T.; Rowan-Nash, A.D.; Sheehan, A.E.; Walsh, R.F.L.; Sanzari, C.M.; Korry, B.J.; Belenky, P. Reductions in anti-inflammatory gut bacteria are associated with depression in a sample of young adults. *Brain Behav. Immun.* **2020**, *88*, 308–324. https://doi.org/10.1016/j.bbi.2020.03.026.
2. Liu, P.; Gao, M.; Liu, Z.; Zhang, Y.; Tu, H.; Lei, L.; Wu, P.; Zhang, A.; Yang, C.; Li, G.; et al. Gut Microbiome Composition Linked to Inflammatory Factors and Cognitive Functions in First-Episode, Drug-Naive Major Depressive Disorder Patients. *Front. Neurosci.* **2022**, *15*, 800764. https://doi.org/10.3389/fnins.2021.800764.
3. Chen, Y.H.; Xue, F.; Yu, S.F.; Li, X.S.; Liu, L.; Jia, Y.Y.; Yan, W.J.; Tan, Q.R.; Wang, H.N.; Peng, Z.W. Gut microbiota dysbiosis in depressed women: The association of symptom severity and microbiota function. *J. Affect. Disord.* **2021**, *282*, 391–400. https://doi.org/10.1016/j.jad.2020.12.143.
4. Hu, X.; Li, Y.; Wu, J.; Zhang, H.; Huang, Y.; Tan, X.; Wen, L.; Zhou, X.; Xie, P.; Olasunkanmi, O.I.; et al. Changes of gut microbiota reflect the severity of major depressive disorder: A cross sectional study. *Transl. Psychiatry* **2023**, *13*, 137. https://doi.org/10.1038/s41398-023-02436-z.
5. Kovtun, A.S.; Averina, O.V.; Angelova, I.Y.; Yunes, R.A.; Zorkina, Y.A.; Morozova, A.Y.; Pavlichenko, A.V.; Syunyakov, T.S.; Karpenko, O.A.; Kostyuk, G.P.; et al. Alterations of the Composition and Neurometabolic Profile of Human Gut Microbiota in Major Depressive Disorder. *Biomedicines* **2022**, *10*, 2162. https://doi.org/10.3390/biomedicines10092162.
6. Rong, H.; Xie, X.H.; Zhao, J.; Lai, W.T.; Wang, M.B.; Xu, D.; Liu, Y.H.; Guo, Y.Y.; Xu, S.X.; Deng, W.F.; et al. Similarly in depression, nuances of gut microbiota: Evidences from a shotgun metagenomics sequencing study on major depressive disorder versus bipolar disorder with current major depressive episode patients. *J. Psychiatr. Res.* **2019**, *113*, 90–99. https://doi.org/10.1016/j.jpsychires.2019.03.017.
7. Lin, S.K.; Chen, H.C.; Chen, I.M.; Hsu, C.D.; Huang, M.C.; Liu, C.M.; Wu, S.I.; Chen, P.Y.; Chen, C.H.; Kuo, P.H. Dysbiosis and depression: A study of gut microbiota alterations and functional pathways in antidepressant-naïve mood disorder patients. *Transl. Psychiatry* **2025**, *15*, 290. https://doi.org/10.1038/s41398-025-03521-1.
8. Hernández-Cacho, A.; García-Gavilán, J.F.; Atzeni, A.; Konstanti, P.; Belzer, C.; Vioque, J.; Corella, D.; Fitó, M.; Vidal, J.; Mela, V.; et al. Multi-omics approach identifies gut microbiota variations associated with depression. *NPJ Biofilms Microbiomes* **2025**, *11*, 68. https://doi.org/10.1038/s41522-025-00707-9.

9. Zu, X.; Xin, J.; Xie, H.; Xu, X.; Shen, Y.; Wang, J.; Tian, S.; Wen, Y.; Li, H.; Yang, J.; et al. Characteristics of gut microbiota and metabolic phenotype in patients with major depressive disorder based on multi-omics analysis. *J. Affect. Disord.* **2024**, *344*, 563–576. https://doi.org/10.1016/j.jad.2023.10.104.
10. Mason, B.L.; Li, Q.; Minhajuddin, A.; Czysz, A.H.; Coughlin, L.A.; Hussain, S.K.; Koh, A.Y.; Trivedi, M.H. Reduced anti-inflammatory gut microbiota are associated with depression and anhedonia. *J. Affect. Disord.* **2020**, *266*, 394–401. https://doi.org/10.1016/j.jad.2020.01.137.
11. Ye, X.; Wang, D.; Zhu, H.; Wang, D.; Li, J.; Tang, Y.; Wu, J. Gut Microbiota Changes in Patients With Major Depressive Disorder Treated With Vortioxetine. *Front. Psychiatry* **2021**, *12*, 641491. https://doi.org/10.3389/fpsyt.2021.641491.
12. Zhang, Q.; Yun, Y.; An, H.; Zhao, W.; Ma, T.; Wang, Z.; Yang, F. Gut Microbiome Composition Associated With Major Depressive Disorder and Sleep Quality. *Front. Psychiatry* **2021**, *12*, 645045. https://doi.org/10.3389/fpsyt.2021.645045.
13. Zhao, H.; Jin, K.; Jiang, C.; Pan, F.; Wu, J.; Luan, H.; Zhao, Z.; Chen, J.; Mou, T.; Wang, Z.; et al. A pilot exploration of multi-omics research of gut microbiome in major depressive disorders. *Transl. Psychiatry* **2022**, *12*, 8. https://doi.org/10.1038/s41398-021-01769-x.
14. Zhou, Y.; Chen, C.; Yu, H.; Yang, Z. Fecal Microbiota Changes in Patients With Postpartum Depressive Disorder. *Front. Cell. Infect. Microbiol.* **2020**, *10*, 567268. https://doi.org/10.3389/fcimb.2020.567268.
15. Tsai, C.F.; Chuang, C.H.; Wang, Y.P.; Lin, Y.B.; Tu, P.C.; Liu, P.Y.; Wu, P.S.; Lin, C.Y.; Lu, C.L. Differences in gut microbiota correlate with symptoms and regional brain volumes in patients with late-life depression. *Front. Aging Neurosci.* **2022**, *14*, 885393. https://doi.org/10.3389/fnagi.2022.885393.
16. Fontana, A.; Manchia, M.; Panebianco, C.; Paribello, P.; Arzedi, C.; Cossu, E.; Garzilli, M.; Montis, M.A.; Mura, A.; Pisanu, C.; et al. Exploring the Role of Gut Microbiota in Major Depressive Disorder and in Treatment Resistance to Antidepressants. *Biomedicines* **2020**, *8*, 311. https://doi.org/10.3390/biomedicines8090311.
